# The fundamental role of causal models in cultural models of nature

**DOI:** 10.3389/fpsyg.2014.01140

**Published:** 2014-10-10

**Authors:** Giovanni Bennardo

**Affiliations:** Anthropology and Cognitive Studies, Northern Illinois UniversityDeKalb, IL, USA

**Keywords:** causality, causal models, cultural models, cultural models of nature, knowledge representations

## Introduction

Cultural Models (CMs) are a way to organize mental knowledge (Holland and Quinn, 1987; Bennardo and De Munck, 2014) within communities of various sizes. Regarding their internal organization, causality plays a major role in CMs' structure/s. A widely accepted way to represent causality in a variety of domains is that of using causal models (Sloman, 2009; Rips, 2011). I propose to think about and use the concept of causal model as a fundamental aspect of CMs. I provide a tentative exemplification of this proposal by looking at causal models in CMs of nature.

## Cultural models and causal models

CMs are assemblages of mental knowledge (i.e., models/representations of the world) shared within a population. CMs function as mental lenses used in understanding, in reasoning, in planning actions, and they may motivate/generate action as well (D'Andrade and Strauss, 1992). CMs are systems, that is, they are constituted by units (e.g., concepts, events, foundational CMs, molar cultural models, etc.) and relationships among these units. Relationships among conceptual units (including CMs of same or different molarity) can be of different types. For example, they can be sequential, taxonomic (also partonomic), and causal.

Sloman affirms that the logic of causality is an invariant of and the best guide to human reasoning and knowledge organization (Sloman, 2009, p. 20). Rips successfully maintains that our ability to infer causality from co-occurrence of events depends on higher-level beliefs, i.e., causal models, about what sort of events can cause others (Rips, 2011, p. 150). He also states that: “Identifying causes requires a healthy dose of theory to direct our search. We can't understand these abstract matters unless the appropriate schemas are already in place” (Rips, 2011, p. 123). In other words, schemas or theories (I prefer the term “model” as in Johnson-Laird, 1980, 1983, 1999) guide and/or generate our causal thinking. Causality, then, appears to be part and parcel of our knowledge of the world while at the same time it depends on knowledge being organized in CMs. This leads to my current proposal of seeing causal models embedded within CMs.

One way in which causality is described and explained is by the use of causal models. First, though, let's see how we can define a cause or a causal relation. Sloman writes that

“A causal relation suggests a mechanism unfolding over time … so the notion of cause involves change over time … [C]ausal relations relate entities that exist in and therefore are bounded in time. I will refer to such entities as *events* or *classes of events* … Causal relations … associate events with other events” [original italics] (Sloman, 2009, pp. 21–22).

If representing the world includes fundamentally the representation of events and if events are mostly associated by causal relations, then, it is these latter that need to be an essential component of CMs.

Causal relations are typically represented by causal models. A causal model consists of (1) a Graph whose input are (2) the World and (3) the Probability Distribution. *The World* consists of the “causal system” we want to represent, a part of the world, e.g., fire, sparks, oxygen, energy source, etc. *The Probability Distribution* consists of the likelihood that certain events (i.e., the content of the World) exist and the likelihood of them going together. For example, while the probability P of fire is typically low, it becomes high when sparks, oxygen, and energy source co-occur, and it becomes zero when there is no oxygen. *The Graph* consists of a representation of the relations among events (i.e., the content of the World) by means of boxes (standing for concepts, events, etc.) and arrows (standing for causal relations). It is the co-presence of oxygen, sparks, and an energy source (3 boxes) that causes (arrow) fire.

Causal Models have been suggested to play a role in reasoning, decision making, judgments, conceptual structure, categorical induction, language, and learning. Relevant to our discussion is the role that they play in conceptual structure since CMs represent organizations of knowledge in conceptual structure.

## Causal models and categorization

Concepts do not only represent sets of objects in the world, but also a set of possible objects. As such they are representing actual and counterfactual objects. This characteristic of concepts is very close to that of causal models insofar as they both can represent possible worlds. Causal relations then can be critical for categorization. Sloman (2009, p. 120) suggests that it would be worth using causal models in exploring relationships not only between events but also between properties of objects.

As an exemplification of this possibility, Sloman introduces a discussion of the theory of function for artifacts proposed by Chaigneau et al. (2004). This theory suggests that the function of an object is related to the following aspects of the same object: its Historical role, the Intentions of an agent using the object, its Physical structure, and the Events that occur when it is used (that is why this theory is also known as the HYPE theory of objects). Finally it is suggested that all these pieces of information are related via a causal model (see Sloman, 2009, p. 122).

The process of categorization that includes a causal model theory of conceptual structure assumes two stages: the first stage relates to some sensory experience; the second stage uses the sensory experience as a cue to retrieve concepts from memory in the form of a causal model (Sloman, 2009, p. 125). For example, one might look at the sky and see what one would interpret to be wings and a body. Then, one also hears a roaring sound.

At first, one may retrieve a causal model in which “has wings,” “has body,” and “has feathers” are related causally to “can fly.” This model though would not fit one's current sensory experience (especially leaving the “roaring sound” unaccounted). In an attempt to account for the roaring sound, one would access a causal model in which “has wheels,” “has body,” and “has engine” would be related causally to a box named “can drive,” while at the same time the “has engine” would cause “makes noise.” The “can drive” box would cause the “can transport people” one. While this model explains the roaring sound by the presence of a causing “engine,” it introduces the concepts of “wheels” and “drive” that are not present in one's current sensory experience.

Finally, a causal model is accessed and activated in which “has wings,” “has body,” and “has engines” cause a box named “can fly” while the “has engines” box by itself causes “makes loud noise.” The “can fly” box would also be causally related to a “can transport people” one. This last model is a good fit for one's sensory experience and provides a plausible causal explanation of all the elements one sensed (Sloman, 2009, p. 126).

Objects may be associated with a number of causal models and the one that is activated depends on people's intentions in dealing with them as well as on the context within which people and objects may be located. What remains constant though is the fact that the relevant knowledge about objects is organized as a causal model in spite of the variety of intentions that people may have in specific contexts (Sloman, 2009, p 128). In addition, categorization serves a multitude of purposes and not all of them can be served by referring to causal properties. These latter are paramount when categorization is intended to reveal the reasons why an object exists, what its use is, what its origin is, and how it works.

## Cultural models of nature

I am currently heading a collaborative research project sponsored by NSF about the cross-cultural concept of nature. The project includes 15 scholars collecting data in 15 communities of primary food producers all over the world. We have prepared a methodological protocol that will be used by all of us both to collect and analyze data (Bennardo, 2012). By the end of 2014, all the data will be collected and the analyses should yield a number of preliminary concepts of nature held within the communities. These concepts will be verified by further data collection and a consensus analysis to be conducted in each community the following year.

The concept of nature is a “complex concept” (see Keller and Lehman, 1991) and I prefer to call it a CM. I have already proposed that a fundamental aspect of any CM is that it contains a causal model. In addition, Sloman's (2009) suggestion of the role of causal models in categorization (concept formation) has also convinced me that the concepts/CMs of nature do include causal models as their fundamental constituent element.

Before benefiting from the results of the mentioned project, I have prepared three “hypotheses” of CMs of nature that are structured to include causal models. In other words, the three CMs of nature I am suggesting include three slightly different types of causal models. The examples of CMs of nature I am using are personal renditions of suggestions in Kempton et al. (1995), in Selin (2003), in Atran and Medin (2008), and in Bennardo et al. (2012).

**Figure 1:**
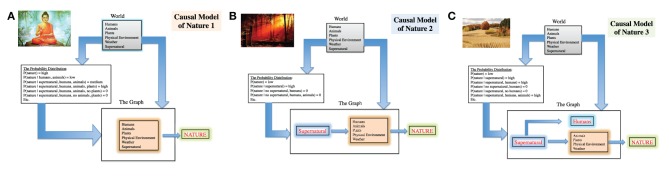
**Three Causal Models of Nature**. **(A)** Holistic, **(B)** Supernatural, and **(C)** Supernatural/Humans.

The first CM of nature can be labeled “holistic” and it is typical of populations whose religion is Buddhism, Confucianism, Shinto, and others (see Selin, 2003). Within this model, all the major elements of the existing world are part of “one” reality which does not have privileged agents (Figure 1A). As such, the causal model in this CM of nature would have these constituent elements in *the World* part: humans, animals, plants, physical environment, weather, and the supernatural. In *the Probability Distribution* part, only a combination in which all the constituent elements of the CM of nature in *the World* part appear receives a high score, while whenever one of the elements is missing, the score would be zero. In *the Graph* part there would be a box with a list of all the elements and an arrow (indicating cause) leading to the concept/CM of nature.

The second CM of nature I called “supernatural” insofar as it implies a separation of a supernatural/creator being from all the other elements in *the World* part of the causal model (Figure 1B). In *the Probability Distribution* the presence of any elements of nature without the supernatural would receive a low or zero score, while the score becomes higher when supernatural and other elements of nature appear together. *The Graph* part of the causal model sees the box of supernatural causing all the other elements, i.e., humans, animals, plants, physical environment, and weather. This causal relationship finally leads to the causation of the concept/CM of nature.

The third CM is labeled “supernatural/humans” because these two elements of *the World* are assigned a privileged position (Figure 1C). The supernatural is still causing all the other elements of the world as in the previous model, but now “humans” are separated both from the “causing” supernatural and from animals, plant, physical environment, and weather. Humans do not cause these latter and they are directly caused by the supernatural.

These causal models—suggested as constituents of different CMs of nature—are only three out of other possible ones. It is important to notice that at *the Probability Distribution* level/part, culture plays a very fundamental role. That is, since perception of the world depends on cultural saliency, the probability distribution of the constituent elements of the concept of nature are dependent on cultural choices. Thus, cultural saliency determines the (perceptual and probable) choices that are then represented in *the (causal) Graph*.

Once the project is completed, we are convinced that we will be able to fill in some of the missing data—especially at *the Probability Distribution* level—that will lead to a refinement of the CMs of nature we are after and their internal causal models. I believe that the introduction of causal models in CM theorizing provides a suitable way to enhance our cross-cultural understanding of the mental organization of knowledge.

### Conflict of interest statement

The author declares that the research was conducted in the absence of any commercial or financial relationships that could be construed as a potential conflict of interest.

## References

[B1] Atran S. Medin D. L. (2008). The Native Mind and the Cultural Construction of Nature. Cambridge, MA: The MIT Press

[B2] Bennardo G. (2012). Proceedings of Workshop: Cultural Models of Nature and the Environment. Working Papers #1 (Dekalb, IL: Institute for the Study of the Environment, Sustainability, and Energy (ESE) at Northern Illinois University). Available online at: http://www.niu.edu/ese/

[B3] Bennardo G. De Munck V. (2014). Cultural Models: Genesis, Methods, and Experiences. Oxford: Oxford University Press

[B4] Bennardo G. Rangel M. Valasek C. LoSavio J. (2012). An american cultural model of nature: a pilot study in northern illinois, in Proceedings of Workshop: Cultural Models of Nature and the Environment, Working Papers #1, ed Bennardo G. (Dekalb, IL: Institute for the Study of the Environment, Sustainability, and Energy (ESE) at Northern Illinois University), 135–142 Available online at: http://www.niu.edu/ese/

[B5] Chaigneau S. E. Barsalou L. W. Sloman S. A. (2004). Assessing affordance and intention in the HIPE theory of function. J. Exp. Psychol. 133, 601–625 10.1037/0096-3445.133.4.601 15584809

[B6] D'Andrade R. G. Strauss C. (eds.). (1992). Human Motives and Cultural Models. Cambridge: Cambridge University Press

[B7] Holland D. Quinn N. (eds.). (1987). Cultural Models in Language and Thought. Cambridge: Cambridge University Press

[B8] Johnson-Laird P. N. (1980). Mental models in cognitive science. Cogn. Sci. 4, 71–115

[B9] Johnson-Laird P. N. (1983). Mental Models: Towards a Cognitive Science of Language, Inference, and Consciousness. Cambridge, MA: Harvard University Press

[B10] Johnson-Laird P. N. (1999). Mental Models, in The MIT Encyclopedia of the Cognitive Sciences, eds Wilson R. A. Keil F. C. (Cambridge, MA: The MIT Press), 525–527

[B11] Keller J. W. D. Lehman F. K. (1991). Complex Concepts. Cogn. Sci. 15, 271–291

[B12] Kempton W. Boster S. J. Hartley J. A. (1995). Environmental Values in American Culture. Cambridge, MA: The MIT Press

[B13] Rips L. J. (2011). Lines of Thought: Central Concepts in Cognitive Psychology. Oxford: Oxford University Press 10.1093/acprof:oso/9780195183054.001.0001

[B14] Selin H. (ed.). (2003). Nature Across Cultures: Views of Nature and the Environment in Non-Western Cultures. Dordrecht, The Netherlands: Kluwer Academic Publishers 10.1007/0-306-48094-8

[B15] Sloman S. A. (2009). Causal Models: How People Think About the World and its Alternatives. Oxford: Oxford University Press

